# FTO Gene Associated Fatness in Relation to Body Fat Distribution and Metabolic Traits throughout a Broad Range of Fatness

**DOI:** 10.1371/journal.pone.0002958

**Published:** 2008-08-13

**Authors:** Sofia I. I. Kring, Claus Holst, Esther Zimmermann, Tine Jess, Tina Berentzen, Søren Toubro, Torben Hansen, Arne Astrup, Oluf Pedersen, Thorkild I. A. Sørensen

**Affiliations:** 1 Institute of Preventive Medicine, Copenhagen University Hospitals, Centre for Health and Society, Copenhagen, Denmark; 2 Center for Pharmacogenomics, the Panum Institute, University of Copenhagen, Copenhagen, Denmark; 3 Reduce–Research Clinic of Nutrition, Hvidovre University Hospital, Hvidovre, Denmark; 4 Steno Diabetes Center, Copenhagen, Denmark; 5 Faculty of Health Science, University of Aarhus, Aarhus, Denmark; 6 Institute of Human Nutrition, Faculty of Life Sciences, University of Copenhagen, Copenhagen, Denmark; Baylor College of Medicine, United States of America

## Abstract

**Background:**

A common single nucleotide polymorphism (SNP) of *FTO* (rs9939609, T/A) is associated with total body fatness. We investigated the association of this SNP with abdominal and peripheral fatness and obesity-related metabolic traits in middle-aged men through a broad range of fatness present already in adolescence.

**Methodology/Principal Findings:**

Obese young Danish men (n = 753, BMI≥31.0 kg/m^2^) and a randomly selected group (n = 879) from the same population were examined in three surveys (mean age 35, 46 and 49 years, respectively). The traits included anthropometrics, body composition, oral glucose tolerance test, blood lipids, blood pressure, fibrinogen and aspartate aminotransferase. Logistic regression analysis was used to assess the age-adjusted association between the phenotypes and the odds ratios for the *FTO* rs9939609 (TT and TA genotype versus the AA genotype), for anthropometrics and body composition estimated per unit z-score. BMI was strongly associated with the AA genotype in all three surveys: OR = 1.17, p = 1.1*10^−6^, OR = 1.20, p = 1.7*10^−7^, OR = 1.17, p = 3.4*10^−3^, respectively. Fat body mass index was also associated with the AA genotype (OR = 1.21, p = 4.6*10^−7^ and OR = 1.21, p = 1.0*10^−3^). Increased abdominal fatness was associated with the AA genotype when measured as waist circumference (OR = 1.21, p = 2.2*10^−6^ and OR = 1.19, p = 5.9*10^−3^), sagittal abdominal diameter (OR = 1.17, p = 1.3*10^−4^ and OR = 1.18, p = 0.011) and intra-abdominal adipose tissue (OR = 1.21, p = 0.005). Increased peripheral fatness measured as hip circumference (OR = 1.19, p = 1.3*10^−5^ and OR = 1.18, p = 0.004) and lower body fat mass (OR = 1.26, p = 0.002) was associated with the AA genotype. The AA genotype was significantly associated with decreased Stumvoll insulin sensitivity index (OR = 0.93, p = 0.02) and with decreased non-fasting plasma HDL-cholesterol (OR = 0.57, p = 0.037), but not with any other of the metabolic traits. However, all significant results for both body fat distribution and metabolic traits were explained by a mediating effect of total fat mass.

**Conclusion:**

The association of the examined *FTO* SNP to general fatness throughout the range of fatness was confirmed, and this association explains the relation between the SNP and body fat distribution and decreased insulin sensitivity and HDL-cholesterol. The SNP was not significantly associated with other metabolic traits suggesting that they are not derived from the general accumulation of body fat.

## Introduction

The human body fatness and the extreme phenotype, obesity, are influenced by both genes and environment as clearly and consistently demonstrated in multiple family, twin and adoption studies [1]. Numerous investigations have been undertaken to further specify these influences. Since the discovery of the leptin gene in 1994 [2], there has been an intense search for obesity genes, but with limited success [1], [3]. Until recently, the only gene in which multiple different, but rare variants have been consistently associated with monogenic fatness is the gene of the melanocortin receptor 4 *(MC4R)* but these mutations are infrequent in the general population [4].

In 1999, a gene was found to be associated with fused-toes in mice and was named fatso *(FTO)* [5]. Recently, a human genome-wide search for type 2 diabetes susceptibility genes identified a common variant (T/A) with a minor allele frequency of 0.45 in the first intron of the *FTO* gene on chromosome 16q12.2 that predisposes to type 2 diabetes through an effect on BMI in Caucasians [6]–[8]. The association was replicated in 13 cohorts with in total 38,759 participants from UK and Finland (p = 3*10^−35^) [6]. The 16% of adults who had the AA genotype for the *FTO* rs9939609 weighed about 3 kg more and had a 1.67 fold increased odds of obesity compared with the non-carriers (TT genotype). In a subset of children aged 9 years, a DEXA scan was conducted and showed that the A allele was primarily associated with the size of the fat mass rather than the lean body mass. The general findings, though with other SNPs in this gene, were replicated in series of other European populations [7]. Two following genome-wide association studies confirmed that the association between *FTO* and type 2 diabetes was entirely mediated by the effect of *FTO* on fatness [8], [9]. This discovery of the consistent association with human fatness has lead to a changed name of *FTO* (GenBank accession no.: NT_010498) from fatso to “fat mass and obesity associated” gene. The function and pathway of the *FTO* gene are unknown, but gene expression profiles show that *FTO* is expressed in particular in specific parts of the brain, muscle and adipose tissue [6], [7]. However, a recent gene expression study suggests that intronic *FTO* SNPs may exert functional effects through altered expression of *FTO* mRNA, particularly in the hypothalamus [10].

We investigate the effects of the *FTO* rs9939609 on different abdominal and peripheral fatness phenotypes and obesity-related metabolic quantitative traits in middle-aged men throughout a very broad range of fatness present already in their adolescence. Further, we examine whether fatness associated with the *FTO* SNP is related to other related metabolic quantitative traits.

## Methods

### Study population

The study population consisted of Danish men originally identified from the mandatory draft board examinations of approximately 360 000 men at a median age of 19 years in the metropolitan area of Copenhagen and surrounding counties from the years 1943–77. Two groups were manually selected in the late 1970'es from this population: one group of all obese men with a BMI≥31 kg/m^2^, n = 1930, (corresponding to 35 % overweight according to a national standard scale in use when the obese sample was identified) and a randomly selected control group consisting of 1% of all men in the study population, n = 3601. The overall prevalence of obesity as defined was thus 1930/360,000 = 0.54 %, which means that all obese were above the 99^th^ percentile of BMI in this population.

Fatness-related phenotypic information was available from three subsequent follow-up surveys of subsets of these two groups: the Copenhagen City Heart Study, 2^nd^ and 3^rd^ survey (year 1981–83 and 1992–94) and the latest follow-up in year 1998–2000. The criteria for invitation to the follow-up surveys and the participation have been described previously [11]–[13], and the number of participants shows the expected attrition over time **(** **Table 1** **).** In the present study, the mean ages at the Copenhagen City Heart Studies were 35 and 46 years, respectively, and the mean age at the last survey was 49 years. In the present study these three surveys are therefore labelled S-35, S-46 and S-49, respectively. Phenotypic assessments were carried out at all three surveys, and DNA was sampled from blood sample buffy coats at the S-46.

**Table 1:** Distribution of age, anthropometrics, metabolic traits and OGTT-derived indices for *FTO* rs9939609 genotyped participants given as median and range.

	S-35		S-46		S-49*	
Variables	*Obese N = 582*	*Control N = 745*	*Obese N = 753*	*Control N = 879*	*Obese N = 231*	*Control N = 320*
Age (yrs)	32.0 [22.0–62.0]	36.0 [22.0–62.0]	43.0 [33.2–72.9]	47.0 [33.1–73.3]	47.0 [39.0–64.0]	49.0 [39.0–65.0]
*Anthropometrics*						
BMI (kg/m^2^)	33.3 [18.4–54.0]	24.5 [16.3–40.4]	35.0 [19.9–63.7]	25.7 [16.2–45.1]	35.3 [23.2–56.4]	25.7 [17.9–42.9]
FBMI (kg/m^2^)	-	-	12.0 [3.4–32.1]	6.3 [0.35–19.2]	11.7 [3.8–25.7]	5.7 [1.1–15.9]
Waist (cm)	-	-	116.5 [79.5–183.0]	93.0 [58.0–139.0]	117.0 [88.0–164.0]	93.0 [69.0–127.0]
SAD (cm)	-	-	27.2 [16.5–52.6]	21.4 [13.8–36.4]	27.3 [18.8; 40.0]	20.8 [15.2; 31.9]
IAAT (cm^2^)	-	-	-	-	182.8 [46.6–308.7]	104.8 [23.0–226.7]
Hip (cm)	-	-	113.9 [48.0–165.0]	100.0 [60.0–132.0]	112.0 [92.0; 155.0]	97.0 [74.0; 126.0]
LBFM (%)	-	-	-	-	29.1 [12.0; 51.2]	18.6 [4.6; 41.4]
*Insulin-glucose related traits*						
Glucose (mmol/L)	5.9 [3.6–22.5]	5.9 [3.3–22.0]	4.0 [2.1–19.6]	3.2 [1.7–11.9]	5.9 [4.5–19.1]	5.6 [4.3–12.8]
Insulin (pmol/L)	-	-	-	-	59.8 [12.0–332.5]	30.5 [6.5–193.0]
C-peptide (mmol/L)	-	-	-	-	808 [272–2418]	554 [173–2107]
HbA1C (%)	-	-	-	-	5.7 [4.7–11.9]	5.6 [4.4–10.1]
Stumvoll index	-	-	-	-	5.2 [−13.0–9.5]	8.8 [−0.5–11.9]
Matsuda index	-	-	-	-	3.6 [0.6–21.1]	6.8 [1.3–32.4]
BIGTT- S_I_	-	-	-	-	1.3 [−3.7–2.5]	2.1 [−0.4–2.8]
BIGTT-AIR	-	-	-	-	7.8 [2.8–10.2]	7.4 [5.1–8.7]
*Other metabolic traits*						
Cholesterol (mmol/L)	5.1 [2.3–8.5]	5.1 [3.0–10.1]	5.9 [1.7–21.8]	6.1 [3.2–17.8]	5.5 [2.4–8.7]	5.7 [3.6–9.8]
Triglycerides (mmol/L)	-	-	-	-	1.6 [0.6–13.2]	1.2 [0.4–8.0]
HDL (mmol/L)	0.9 [0.3–1.8]	1.0 [0.3–2.1]	1.1 [0.3–3.0]	1.4 [0.5–3.9]	1.0 [0.5–2.0]	1.2 [0.6–2.4]
FFA (mmol/L)	-	-	-	-	0.4 [0.1–1.1]	0.4 [0.1–0.9]
Fibrinogen (g/L)	-	-	320.0 [3.0–858.0]	275.0 [135.0–3004.0]	-	-
ASAT (IU/L)	-	-	24.0 [7.0–280.0]	23.0 [3.0–530.0]	-	-
Systolic BP (mmHg)	138.0 [103.0–213.0]	132.0 [95.0–211.0]	143.0 [103.0–247.0]	136.0 [101.0–219.0]	129.0 [80.0–194.0]	122.0 [90.0–194.0]
Diastolic BP (mmHg)	88.0 [49.0–148.0]	82.0 [47.0–130.0]	95.0 [58.0–151.0]	90.0 [62.0–128.0]	80.0 [57.0–121.0]	76.0 [54.0–114.0]

BMI = body mass index, FBMI = fat body mass index, IAAT = intra-abdominal adipose tissue, SAD = sagittal abdominal diameter, LBFM (%) = lower body fat mass (%), SAD = Sagittal abdominal diameter, BP = blood pressure, FFA = free fatty acids, BIGTT-S_I_ = OGTT-derived index of insulin sensitivity, BIGTT-AIR = OGTT-derived index of acute insulin response. ^*^ All values for the S-49 are derived from the OGTT examination and are therefore fasting compared to non-fasting for S-35 and S-46. The metabolic traits except for BP were derived from plasma blood samples

### Phenotypic measurements

Table 1 serves descriptive purposes for the three study samples and lists the fatness measures and obesity-related metabolic phenotypes available for analysis from S-35, S-46 and S-49, and the basic statistics (median and range) in the obese and control groups. Total body fat mass (kg) was assessed by bioimpedance at S-46 and from the DEXA scan at S-49. Fat body mass index (FBMI; kg/m^2^) was calculated as total body fat mass (kg) divided by height (m) squared. Intraabdominal adipose tissue (IAAT; cm^2^) was calculated from DEXA scans and anthropometry using the equation [14]: y = −208.2+4.62 (sagittal diameter, cm)+0.75 (age, y)+1.73 (waist, cm)+0.78 (trunk fat, %). In S-46 and S-49 sagittal abdominal diameter (SAD, cm) was measured with the participant in expiration phase and lying recumbent on an examination table, as the distance between the top of the examination table and a horizontally placed spirit level placed above the abdomen at the level of the iliac crest. Lower body fat mass (LBFM; %) was calculated from DEXA scans as the fat percentage of lower body fat mass. All body composition measurements, except for waist and hip circumference in S-46 are derived from bioimpedance and in S-49 derived from DEXA-scans. The bioimpedance method has proved to be reasonably accurate for assessment of body composition (fat free mass and fat mass) [15] and the present results are not influenced by the fact that separate prediction equations are necessary for different ethnic groups [16]. However, measurement of total body fat by DEXA-scans are more accurate than by the impedance method [15].

Participants in S-35 and S-46 had non-fasting glucose levels determined on fresh plasma samples. In the S-49 cohort oral glucose tolerance tests (OGTT) were conducted but with the exclusion of individuals with known *diagnosed* and thereby treated diabetes (n = 10) [11]. Furthermore, we also derived indices of insulin sensitivity according to Stumvoll [17], Matsuda [18], and the recently recommended BIGTT index (BIGTT-S_I_) [19]. An index for insulin secretion was derived (BIGTT-AIR (acute insulin response)). For BIGTT-S_I_ and BIGTT-AIR the measurements of plasma glucose and serum insulin at the time points 0, 30 and 120 minutes during the OGTT were used. Details on data collections and measurement of anthropometric and other phenotypic estimates have been described elsewhere [11], [13], [20], [21].

### Molecular genetic analyses

Genotyping of the *FTO* rs9939609 SNP was performed using Taqman allelic discrimination (KBiosciences, Herts, UK). Genotype data were obtained in more than 97% of the DNA samples with a genotype error rate of 0.27% based on 1464 duplicate samples. All genotype groups obeyed Hardy-Weinberg equilibrium and the minor allele frequency was 0.41–0.42 in controls and 0.51–0.52 in obese individuals. Molecular genetic analysis, including genotyping of the *FTO* SNP rs9939609, was conducted on 879 controls and 753 cases.

### Statistical analysis

In a logistic regression analysis we tested the effect of rs9939309 on having a BMI≥31.0 kg/m^2^ at the draft board examination with the TT genotype as the reference group; we found that the odds ratio (OR) for the TA genotype was 1.21 [0.96–1.52] and for the AA genotype 2.04 [1.54–2.70]. A likelihood ratio test (LRT) for an additive co-dominant effect of the gene was LRT = 2.76, p = 0.0969, a dominant effect of the gene LRT = 16.55, p<0.0001 and a recessive effect of the gene LRT = 2.62, p = 0.1052. On basis of these tests, which show that a recessive model is most compatible with our data, we have chosen a recessive transmission mode for the present analyses (TT and TA genotype versus AA genotype). In order to properly take into account the sampling design, the two groups of obese and controls have been analysed together, but separately for each follow-up survey S-35, S-46 and S-49. Thus, the massive enrichment of the right tail of the BMI distribution implies that the data cannot be analysed with BMI or BMI-associated outcomes as response variables in common regression models. However, to take advantage of the greater statistical power and much wider coverage of the phenotypes by keeping the obese and non-obese groups in the analysis, we reversed the statistical models for the associations and examined the probability of the particular genotypes for a given level of the phenotypes. This can be done without distributional assumptions about the phenotypes. Hence, logistic regression analysis was used to assess the odds ratios of the genotype (response variable) in relation to the phenotypes (covariates) in the combined case and control groups. The response variable was the AA genotype versus AT and TT genotypes. The other covariates were each phenotype as measured at each of the follow-up surveys (table 1) with and without adjustment for the concurrent FBMI (kg/m^2^). Information for FBMI was not available in S-35 and therefore we adjusted for BMI in these analyses.

To obtain similar fatness units, all anthropometrics and body composition measures were converted to age-adjusted z-scores, which indicate the deviations from the population mean values in standard deviation (SD) units. The age-adjusted z-scores were calculated from the mean values and SD of the randomly selected control group and applied to the entire study population. This conversion into z-scores enables us to directly compare the strength of the association between the genotype and the various fatness phenotypes. In the remainder analyses, age at examination was included as covariate. Smoothing splines with 5 degrees of freedom in general additive models (GAM) were used to assess and test for linearity. Significance level was accepted at p<0.05. Analyses were carried out with SAS statistical procedures (version 9.1; SAS Institute Inc, Cary, NC) and STATA (version 9.2; Stata Corporation, College Station, Texas).

### Ethics

The Danish surveillance Agency and the regional Ethical Committee approved the study to be in accordance with the Helsinki Declaration II. All participants signed a written consent before participating.

## Results

### Descriptive analysis

**Figure 1:**
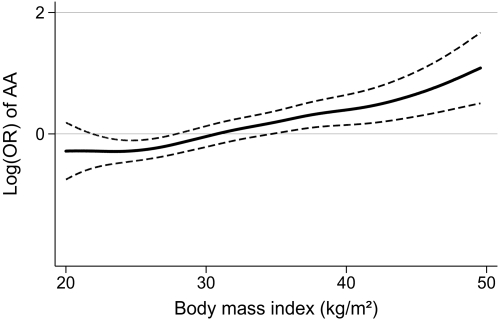
Smoothing spline (5 degrees of freedom) with 95% confidence limits of the association between the FTO rs9939609 AA genotype and BMI (kg/m^2^) at S-46 as assessed by logistic regression with the odds of the AA genotype as the response variable and the BMI as covariate.

The distribution of the *FTO* rs9939609genotypes were almost exactly the same at the three surveys indicating that the attrition of the study groups by time was not dependent on the genotype **(** **Table 2** **)**. There was a linear association with the odds ratio of being carrier of the *FTO* rs9939609 AA genotype throughout the broad range of BMI and FBMI **(** **Figure 1** **and** **2** **)**.

**Figure 2:**
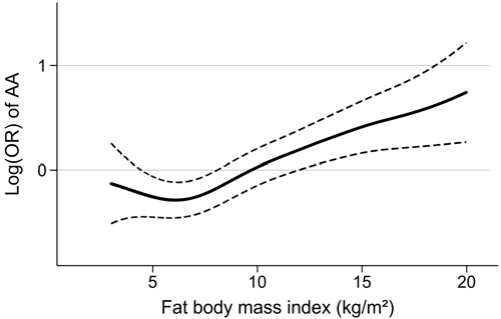
Smoothing spline (5 degrees of freedom) with 95% confidence limits of the association between the FTO rs9939609 AA genotype and fat body mass index (FBMI; kg/m^2^) at S-46 as assessed by logistic regression with the odds of the AA genotype as the response variable and the FBMI as covariate.

**Table 2:** Genotype distribution of *FTO* rs9939609 for controls and obese participants in S-35, S-46 and S-49, respectively

Survey	Controls				Obese			
	*TT (%)*	*TA (%)*	*AA (%)*	MAF	*TT (%)*	*TA (%)*	*AA (%)*	MAF
S-35	241 (32.4)	380 (51.0)	124 (16.6)	0.42	140 (24.1)	280 (48.1)	162 (27.8)	0.52
S-46	287 (32.7)	444 (50.5)	148 (16.8)	0.42	192 (25.5)	359 (47.7)	202 (26.8)	0.51
S-49	114 (35.6)	151 (47.2)	55 (17.2)	0.41	61 (26.4)	104 (45.0)	66 (28.6)	0.51

MAF = Minor allele frequency

### Analysis of fatness

We estimated the odds ratios (OR) of the genotype (response variable) in relation to the phenotypes (covariates). Results from logistic regression analyses for fatness phenotypes are given in z-score odds ratios with 95% confidence intervals. The OR for being carrier of the AA genotype according to BMI z-score should be interpreted as an increment in odds for being carrier of the AA genotype per unit increase in BMI z-score. One z-score unit is equal to 1 SD of the particular trait in the control sample, and for the present quantification we have provided the original unit value for all fatness phenotypes.

The association between the AA genotype for rs9939609 and BMI was strongly confirmed in the three surveys at S-35 (OR = 1.17, p = 1.1*10^−6^), S-46 (OR = 1.20, p = 1.7*10^−7^) and S-49 (OR = 1.17, p = 3.4*10^−3^) **(** **Table 3** **)**. In e.g. S-49 an increase in one unit of z-score BMI, equivalent to an increase in 3.7 kg/m^2^, increased the odds for the AA genotype by 17%. FBMI was strongly associated with the AA genotype, as measured by bioimpedance at S-46 (OR = 1.21, p = 4.6*10^−7^) and DEXA scan at S-49 (OR = 1.21, p = 0.001).

**Table 3:** Odds ratio (OR) including 95% confidence intervals (CI) for *FTO* rs9939609 in relation to fatness phenotypes (in z-scores) including original unit value of 1 SD

Variables	S-35 *N = 1327*			S-46 *N = 1632*			S-49 *N = 551*		
	Original unit	OR (95% CI)	P-value	Original unit	OR (95% CI)	P-value	Original unit	OR (95% CI)	P-value
*z-score of:*									
BMI (kg/m^2^)	3.06	1.17 [1.10; 1.25]	1.1*10^−6^	3.7	1.20 [1.12; 1.29]	1.7*10^−7^	3.7	1.17 [1.05; 1.30]	3.4*10^−3^
FBMI (kg/m^2^)		-	-	2.4	1.21 [1.12; 1.30]	4.6*10^−7^	2.6	1.21 [1.08; 1.36]	1.0*10^−3^
Waist (cm)		-	-	10.5	1.21 [1.12; 1.31]	2.2*10^−6^	10.7	1.19 [1.05; 1.34]	5.9*10^−3^
SAD (cm)		-	-	3.3	1.17 [1.08; 1.27]	1.3*10^−4^	3.3	1.18 [1.04; 1.34]	1.1*10^−2^
IAAT (cm^2^)		-	-	-	-	-	38.1	1.21 [1.06; 1.39]	4.7*10^−3^
Hip (cm)		-	-	6.8	1.19 [1.10; 1.29]	1.3*10^−5^	6.8	1.18 [1.05; 1.31]	3.5*10^−3^
LBFM (%)		-	-	-	-	-	6.2	1.26 [1.09; 1.45]	1.6*10^−3^

BMI = body mass index, FBMI = fat body mass index, IAAT = intra-abdominal adipose tissue, SAD = sagittal abdominal diameter, LBFM (%) = lower body fat mass (%)

Further, increased abdominal fatness was associated with the AA genotype measured as waist circumference (OR = 1.21, p = 2.2*10^−6^ and OR = 1.19, p = 5.9*10^−3^), sagittal abdominal diameter (OR = 1.17, p = 1.3*10^−4^ and OR = 1.18, p = 0.011) and intra-abdominal adipose tissue (OR = 1.21, p = 0.005). Increased peripheral fatness measured as hip circumference (OR = 1.19, p = 1.3*10^−5^ and OR = 1.18, p = 0.004) and LBFM (%) (OR = 1.26, p = 0.002) was also associated with the AA genotype.

### Analysis of insulin-glucose related traits

The associations between the *FTO* gene variant, and glucose, insulin, C-peptide, and HbA1C were not statistically significant **(** **Table 4** **)**.

**Table 4:** Odds ratio (OR) including 95% confidence intervals (CI) for *FTO* rs9939609 in relation to metabolic traits and OGTT-derived indices

Variables	S-35		S-46		S-49*	
	OR (95% CI)	*P*	OR (95% CI)	*P*	OR (95% CI)	*P*
*Insulin-glucose related traits*						
Glucose (10 mmol/L)	0.88 [0.33; 2.34]	0.79	1.01 [0.97; 1.05]	0.69	0.89 [0.20; 3.97]	0.87
Insulin (50 pmol/L)	-	-	-	-	1.08 [0.86; 1.35]	0.49
C-peptide (500 mmol/L)	-	-	-	-	1.18 [0.90; 1.54]	0.24
HbA1C (%)	-	-	-	-	0.94 [0.71; 1.26]	0.69
Stumvoll index	-	-	-	-	0.93 [0.88; 0.99]	0.02
Matsuda index	-	-	-	-	0.97 [0.92; 1.02]	0.22
BIGTT-S_I_	-	-	-	-	0.83 [0.67; 1.03]	0.09
BIGTT-AIR	-	-	-	-	1.36 [0.97; 1.90]	0.08
*Other metabolic traits*						
Cholesterol (10 mmol/L)	2.29 [0.63; 8.32]	0.21	2.18 [0.94; 5.09]	0.07	2.90 [0.44; 19.2]	0.27
Triglycerides (mmol/L)	-	-	-	-	1.05 [0.89; 1.23]	0.56
HDL (mmol/L)	0.57 [0.34; 0.97]	0.037	0.81 [0.60; 1.08]	0.15	0.53 [0.25; 1.10]	0.09
FFA (mmol/L)	-	-	-	-	3.02 [0.85; 10.8]	0.09
Fibrinogen (10 g/L)	-	-	1.01 [1.00; 1.02]	0.11	-	-
ASAT (100 IU/L)	-	-	1.52 [0.83; 2.78]	0.17	-	-
Systolic BP (10 mmHg)	0.99 [0.91; 1.09]	0.89	1.02 [0.96; 1.09]	0.56	1.00 [0.89; 1.12]	0.95
Diastolic BP (10 mmHg)	1.01 [0.91; 1.13]	0.79	1.00 [0.90; 1.11]	0.94	1.06 [0.88; 1.28]	0.54

HbA1C = glycated hemoglobin, BIGTT-S_I_ = OGTT-derived index of insulin sensitivity, BIGTT-AIR = OGTT-derived index of acute insulin response

* All values for the S-49 are derived from the OGTT examination and are therefore fasting compared to non-fasting for S-35 and S-46

Except for a decreased Stumvoll index for insulin sensitivity (OR = 0.93, p = 0.02) none of the remainder OGTT-derived indices were significantly associated with the FTO AA genotype. Albeit not significant, the Matsuda index and the novel BIGTT-S_I_ test for insulin sensitivity showed similar association as the Stumvoll index. Insulin release assessed through the novel BIGTT-AIR test was increased for individuals with the AA genotype, but only borderline significant (OR = 1.36, p = 0.08).

### Analysis of other metabolic traits

Cholesterol and triglycerides levels were not significantly associated with the AA genotype. However, a decreased plasma HDL-cholesterol (OR = 0.57 per mmol/L, p = 0.037) level was seen in individuals with the AA genotype when assessed in S-35 (Table 4). Similar associations were seen at following assessments but with borderline significant results.

Free fatty acids, fibrinogen and ASAT were not significantly associated with the *FTO* SNP. Finally, neither systolic nor diastolic blood pressure was significantly associated with the *FTO* SNP.

### Analysis adjusting for FBMI

The statistical significance of the association between the *FTO* genotype and waist circumference, sagittal abdominal diameter, hip circumference, HDL-cholesterol and Stumvoll index for insulin sensitivity all vanished when the regression analysis adjusted the association for FBMI, whereas the association between the genotype and the FBMI was maintained **(** **Table 5** **).**

**Table 5:** Odds ratio (OR) including 95% confidence intervals (CI) for *FTO* rs9939609 in relation to selected fatness phenotypes and metabolic traits (adjusted variables) and fat body mass index (FBMI; kg/m^2^) (adjusting variable)

Variables	OR (95% CI)	*P*	OR (95% CI)	*P*
	Adjusted		FBMI	
Waist (cm)	0.97 [0.75; 1.28]	0.86	1.24 [0.97; 1.57]	0.09
SAD (cm)	0.87 [0.72; 1.06]	0.17	1.34 [1.13; 1.58]	0.0006
Hip (cm)	0.98 [0.83; 1.18]	0.88	1.22 [1.05; 1.43]	0.01
HDL (mmol/L) at S-46	0.98 [0.68; 1.42]	0.92	1.21 [1.11; 1.31]	<0.0001
HDL (mmol/L) at S-49	0.88 [0.39; 1.97]	0.75	1.21 [1.06; 1.37]	0.004
Stumvoll index	1.03 [0.92; 1.15]	0.59	1.29 [1.04; 1.59]	0.02

All variables are given for S-46, except for HDL-cholesterol, which is given for S-46 and S-49 and Stumvoll index, which is given forS-49.

## Discussion

Results from the present study show that the AA genotype of the examined SNP in the *FTO* gene contributes to human fatness irrespective of adipose tissue distribution. This study adds to establish the more precise nature of the metabolic effects of the recently described fat mass and obesity associated gene variant. Firstly, we have demonstrated associations between *FTO* rs9939609 and insulin sensitivity and plasma HDL-cholesterol levels and secondly, demonstrated that these associations are explained by the mediating effect of FBMI and thirdly, we imply that the *FTO*-related fatness may not be the type of fatness that is associated with the remainder examined metabolic traits, which may be dependent on other additional determinants, e.g. genetic predisposition and chronic inflammation in the adipose tissue.

The present study confirms the initial reports of a positive association between *FTO* rs9939609 and BMI throughout its range. Our results showed a relatively large effect of the genotype on the examined phenotypes. Thus, the function of *FTO* appears to be highly physiologically relevant for the understanding of the pathogenesis of obesity, and this may also have health-related significance. For example at S-49, an increase in one unit of z-score BMI and z-score waist, equivalent to an increase in 3.7 kg/m^2^ and 10.7 cm, respectively, was associated with 17–19% increase in odds for the AA genotype. In a recent large population-based study of approximately 276,835 children, surprisingly little increase in body weight throughout the range of body weights was associated with increases in the risk of coronary heart disease in adulthood [22]. Owing to the sampling design of the obese participants with massive enrichment of the right tail of the BMI distribution it is possible to demonstrate a stronger association compared to already published studies examining the effects of *FTO* on fatness [6], [7]. The present regression analysis with the genotype as response variable and the phenotypes as covariates may be a limitation, albeit the analysis strategy is conditional on the strength of the sampling design. The statistical analysis serves the investigation of the possible association in occurrence of genotype and phenotype, and if there is evidence for an association, then the next phase is the putative causal interpretation, which goes on independent of the statistical modelling.

Population stratification may occur due to differences in allele frequencies between cases and controls owing to systematic differences in ancestry rather than association of genes with the response variable. However, the fundamental theorem of Hardy-Weinberg (H-W) law is that in large homogenous, randomly mating populations the probabilities of H-W law are preserved from generation to generation and, further in non-homogenous but randomly mating populations, they are established in a single generation after mixing [23]. In spite of Denmark being a relatively small country with only about 5 million inhabitants, we have no reason to suspect detectable relatedness in our study population; this is also reflected in the genotype distributions of the sample, which obeyed H-W equilibrium. The case-cohort study design of Danish Caucasian men where the controls were randomly selected from the same population in which the cases were identified effectively prevents population stratification.

The availability of several repetitive measurements in the same individuals have given the unique opportunity of analyzing and comparing a panel of specific fatness phenotypes covering BMI, fat mass and abdominal and peripheral fatness at different ages. The samples may seem small for a genetic association study, however, this apparent limitation of the study is counteracted by the fact that the control group represents 184 000 men in S-46 and 64 600 in S-49, originally identified at the draft board examination, and that the obese participants therefore were representing the most extreme range of the fatness phenotypes in this population at all three surveys except for the possible effects of selective attrition of the samples during the follow-up surveys.

The metabolic traits associated with obesity may be inter-correlated to various extents, but according to a recent twin study [24], there is little common underlying genetic or shared environmental etiology behind these correlations, which we think justifies the separate analysis of each of the traits as we have conducted here barring the analyses where we examined if the total body fatness could explain the observed association between *FTO* and other traits.

The *FTO* gene variant appeared to be related to body fat distribution and some related metabolic phenotypic measures. The AA genotype was associated with increased abdominal fatness examined as waist circumference, sagittal abdominal diameter and intra-abdominal adipose tissue mass. The AA genotype was also associated with increased peripheral fatness examined as hip circumference and lower body fat mass. The strength of the associations between *FTO* and the various fatness phenotypes was about equal throughout the range of each phenotype and the associations vanished when the FBMI was adjusted for by the regression analysis, suggesting that the effect of the gene variant is a general increase of the size of the fat mass irrespective of site and size. Similar results were observed in a study of a Canadian group of men and women; this group corresponded in size and sampling frame to our randomly selected control group only, and the study did not examine if the total fat mass could explain the associations with the body fat distribution measures [25].

No significant associations were seen between the *FTO* SNP and any of the metabolic traits related to the glucose homeostasis (plasma glucose, insulin, C-peptide, HbA1C, OGTT-derived indices for insulin sensitivity and release) except for a rather weak inverse association with the Stumvoll index for insulin sensitivity, which disappeared when FBMI was adjusted for. This finding was expected because fatness is inversely correlated with insulin sensitivity [26]. The abovementioned Canadian study also found an association between *FTO* and various measures of insulin sensitivity, which all vanished when adjusted for BMI [25].

Further, except for the inverse association between plasma HDL-cholesterol and the AA genotype, no significant associations were observed for the investigated lipid levels including FFA, liver function variables or blood pressure. HDL-cholesterol levels were decreased in all three surveys, though only significantly in S-35. The present finding is consistent with results from other studies showing decreased plasma HDL-cholesterol levels commonly found in obese individuals [27], and also this association vanished when adjusted for FBMI. The mediating effects of FBMI suggest that the observed increase in fatness is due to a total increase in fat mass rather than abdominal fat accumulation. None of the remainder examined metabolic quantitative traits were associated with *FTO* rs9939609.

This implies that the *FTO*-related fatness does not contribute to the type of fatness that is associated with these metabolic traits. For example, for the widely known association between obesity and blood pressure [13] we did not find a significant association between rs9939609 and blood pressure. This means that *the* type of fatness that rs9939609 causes is not the particular type of fatness that lead to e.g. increased blood pressure. Nevertheless, we are aware of the fact that although the confidence intervals were quite narrow, reflecting the statistical power of the study design, the reported effects sizes attributable to genetic variation may be so small that we may overlook a true association with a related metabolic phenotype (type 2 error).

In order to elucidate the effects of the *FTO* SNP it may be interesting to assess the impact of the SNP on weight dynamics throughout life and general growth during childhood and adolescence. Likewise, the impact of environmental factors (e.g. physical activity and dietary energy intake) on genetic susceptibility needs to be further explored [28], [29]. In conclusion, results from the present study show that examined SNP in *FTO* contributes to human fatness throughout a very broad range with a corresponding, non-differential effect on adipose tissue distribution. Except for decreased insulin sensitivity and HDL-cholesterol, which was explained by the mediating effect of FBMI, the remainder metabolic quantitative traits were not significantly associated with *FTO* rs9939609.
